# Multielemental Profile for Seminal Plasma Through Inductively Coupled Plasma–Tandem Mass Spectrometry and Its Relationship with Seminal Parameters, Spermatic Biomarkers, and Oxidative Stress

**DOI:** 10.3390/antiox14091118

**Published:** 2025-09-15

**Authors:** Andrea López-Botella, Natalia Cenitagoya-Alonso, Raquel Sánchez-Romero, Paula Sáez-Espinosa, Miranda Hernández-Falcó, María José Gómez-Torres, José Luis Todolí-Torró

**Affiliations:** 1 Biotechnology Department, Faculty of Sciences, University of Alicante, Carretera San Vicente del Raspeig s/n, San Vicente del Raspeig, 03690 Alicante, Spain; andrea.lopez@ua.es (A.L.-B.); paula.saez@gcloud.ua.es (P.S.-E.); miranda.hernandez@gcloud.ua.es (M.H.-F.); mjose.gomez@gcloud.ua.es (M.J.G.-T.); 2 Analytical Chemistry Department, Nutrition and Food Sciences, University of Alicante, Carretera San Vicente del Raspeig s/n, San Vicente del Raspeig, 03690 Alicante, Spain; natalia.cenitagoya@ua.es (N.C.-A.); r.sanchez@mscloud.ua.es (R.S.-R.)

**Keywords:** heavy metals, metalloids, human sperm, seminal plasma, male fertility, inductively coupled plasma–tandem mass spectrometry, oxidative stress

## Abstract

The present study investigated the decline in human fertility by analyzing the multielemental profile of seminal plasma and its relationship with seminal parameters and sperm biomarkers. Twenty-nine donor seminal plasma samples were examined using inductively coupled plasma–tandem mass spectrometry (ICP-MS/MS). Method optimization demonstrated that robust plasma conditions, including internal standardization and helium (He) collision gas, were essential to achieve reliable quantification. These conditions mitigated matrix effects and spectroscopic interferences, despite lower sensitivity. Elements such as copper (Cu), iron (Fe), manganese (Mn), strontium (Sr), titanium (Ti), vanadium (V), and chromium (Cr) were quantified, and several significant correlations were identified. Specifically, Cu was negatively correlated with seminal volume and positively correlated with sperm concentration and spontaneous acrosome reacted sperm, but negatively correlated with medium mitochondrial membrane potential (MMP); Mn showed negative associations with sperm vitality and medium MMP; Fe showed a negative correlation with motile sperm concentration (4 h); V was positively correlated with acrosome reacted sperm after acrosome reaction induction and with very low/medium MMP, whereas it was negatively associated with tyrosine phosphorylation; and Cr also showed a negative correlation with tyrosine phosphorylation. As, Mo, and Pb were detected in a few samples, limiting correlation analysis. From a functional perspective, elements such as As and Pb, as well as excess Cu or Fe, may contribute to oxidative stress by enhancing reactive oxygen species (ROS) generation and impairing antioxidant defenses. Conversely, essential metals, including Mn and Cu, at physiological concentrations act as cofactors of antioxidant enzymes and play a protective role against oxidative damage.

## 1. Introduction

A crucial concern in modern developed societies is the continuous decline in human fertility over the past seven decades that appears to be closely linked to environmental contaminant exposure and lifestyle deterioration. Male reproductive function can therefore be impaired by the action of several species, including heavy metals and metalloids [1,2,3]. These species, considered endocrine-disrupting chemicals (EDCs), induce oxidative stress (OS), a key mechanism underlying male infertility [1]. Once inside the body, certain elements tend to accumulate, thereby exacerbating infertility-related problems [4].

Under physiological conditions, reactive oxygen species (ROS) affect the normal sperm function. However, exposure to heavy metals enhances ROS generation while reducing antioxidant defenses, resulting in redox imbalance and OS. This imbalance causes cellular alterations, such as DNA damage, lipid peroxidation, and apoptosis, which ultimately impair semen quality [5,6,7,8]. In this context, patients with elevated ROS production in semen may benefit from antioxidant therapy. For example, a diet rich in carotenoids may improve sperm motility and morphology.

Therefore, exposure to heavy metals and metalloids has been identified as a relevant factor affecting both spermatogenesis and fertility [9,10]. Environmental pollution [11,12], contaminated food sources [4], and occupational exposure [13] are primary contributors to heavy metal accumulation in reproductive tissues. To assess the male reproductive risks, several biological matrices are commonly analyzed, including blood, serum, urine, or hair. Obviously, semen and seminal plasma (SP) are gaining interest for complete and direct characterization of the impact of heavy metals on fertility. Seminal plasma plays a key role in determining semen quality and, consequently, fertilization success [1]. Therefore, the SP analysis constitutes a valuable tool for the assessment of heavy metal concentrations in male reproductive samples.

Up to date, the most widely employed techniques for the determination of heavy metals and metalloids in human seminal plasma are atomic absorption-based techniques, including flame (FAAS), graphite furnace (GF-AAS), hydride generation (HG-AAS), and cold vapor generation (CV-AAS), as well as inductively coupled plasma–optical emission spectroscopy (ICP-OES) and inductively coupled plasma–mass spectrometry (ICP-MS) [14]. Among these techniques, ICP-MS provides extremely low limits of detection and wide dynamic ranges, and it has the capability of determining a long list of elements in a single analysis. Furthermore, it is perfectly suited for the analysis of low amounts of sample. However, non-spectroscopic, as well as spectroscopic, interferences are produced, thus degrading the accuracy of the determinations.

Regarding non-spectroscopic interferences caused by both inorganic and organic concomitants, the use of spectrometric devices such as aerosol phase dilution systems can minimize the impact of saline matrices on analytical results by preventing solid deposition at the ICP-MS interface. In addition, auxiliary approaches, such as internal standardization, are recommended. Organic matter, in turn, may lead to overestimation of analyte concentrations. To avoid this effect, an acid digestion step (e.g., with nitric acid) is commonly employed [15,16]. It is worth mentioning that the matrix composition of seminal plasma includes mostly citrates, sugars (e.g., fructose and glucose), proteins, hormones, lipids, calcium, magnesium, potassium, and sodium.

To address spectroscopic interferences, reaction and collision cells have recently been applied in the analysis of seminal plasma [17]. On this subject, inductively coupled plasma–tandem mass spectrometry (ICP-MS/MS) has emerged as a promising tool for trace multielemental analysis [18]. In ICP-MS/MS, a first quadrupole is located after the spectrometer interface. Then, an octopole collision–reaction cell is used to promote either the removal of polyatomic ions or the reaction of the analyte ions with gases such as hydrogen, oxygen, ammonia, or helium, among others. Subsequently, a second quadrupole is employed to select the ion for final detection. The versatility of this assembly allows for the removal of spectral interferences, thus making it possible to perform accurate determinations in the presence of clinical matrices [19,20]. To the best of the authors’ knowledge, no previous study has applied ICP-MS/MS technology to the direct analysis of diluted SP. Therefore, the main objective of the present study was to develop and optimize a simple dilution-based method for multielement analysis of human seminal plasma. Furthermore, this study aimed to explore the relationships between quantified trace elements in SP and both conventional semen parameters and functional sperm biomarkers, including sperm capacitation, acrosome reaction, and mitochondrial membrane potential, with particular focus on oxidative stress processes potentially influenced by these elements. Additionally, the work sought to identify which elements could serve as informative indicators of male fertility.

## 2. Materials and Methods

### 2.1. Semen Sample Analysis

Seminal samples were collected from 29 donors obtained via masturbation after three to four days of abstinence from sexual activity, collected between February 2022 and February 2023. All donors provided informed consent prior to sample collection. Semen samples were analyzed within one hour of collection. Sperm concentration and motility were determined using a Makler counting chamber (BioCareEurope, Rome, Italy), morphology was evaluated with Papanicolaou staining (Panreac Química S.L.U., Barcelona, Spain), and viability was assessed using Sperm VitalStain™ (NidaCon International AB, Mölndal, Sweden). Seminal plasma was collected after centrifugation for 10 min at 300× *g* and stored at −40 °C until its use, while the cellular fraction was later used.

### 2.2. Sperm Capacitation by Swim-Up

The procedure used for sperm capacitation has been described in previous scientific studies [21,22]. The cellular pellet was first washed using Human Tubal Fluid (HTF; Origio^®^, Måløv, Denmark). Spermatozoa were then incubated in HTF supplemented with 5 mg/mL bovine serum albumin (BSA; Sigma-Aldrich^®^, St. Louis, MO, USA) at 37 °C in a 5.5% CO_2_ atmosphere for 1 h and 4 h. Subsequently, the supernatant was collected and subjected to three washes in sterile-filtered Dulbecco’s phosphate-buffered saline lacking calcium, magnesium, and phenol red (Capricorn Scientific GmbH, Ebsdorfergrund, Germany) by centrifugation at 250× *g* for 10 min. After capacitation, the motile spermatozoa concentration (MSC) was assessed after 1 h (MSC1) and 4 h (MSC4) of in vitro capacitation.

### 2.3. Induction of Acrosomal Reaction

The acrosome reaction (AR) was induced by incubating the sperm with 10 μM calcium ionophore A23187 (Sigma-Aldrich^®^, St. Louis, MO, USA) and 2 mM calcium chloride (Panreac Química S.L.U., Barcelona, Spain) at 37 °C in a 5.5% CO_2_ atmosphere for 1 h, following established protocols [23].

### 2.4. Fixation

Sperm cells from the different physiological conditions were fixed before capacitation by swim-up (NC), after sperm capacitation (C 1 h and C 4 h), and after the induction of acrosome reaction next to sperm capacitation (AR 1 h and AR 4 h). All samples were initially fixed in 2% (*w*/*v*) paraformaldehyde (Electron Microscopy Sciences, Hatfield, PA, USA) prepared in PBS for 45 min at 4 °C. Following fixation, the paraformaldehyde solution was removed and replaced with PBS to standardize the sperm concentration to 10 million cells per milliliter. Samples were then stored at 4 °C until further analysis.

### 2.5. Evaluation of Acrosomal Reaction

To verify acrosome reaction induction, 5 μL of each sample was fixed onto coverslips with methanol for 30 min. Following three washes with PBS, samples were incubated with Pisum sativum agglutinin conjugated to fluorescein-5-isothiocyanate (PSA-FITC; Sigma-Aldrich^®^, St. Louis, MO, USA) at a final concentration of 50 μg/mL for 30 min, and then washed three times with PBS. Finally, the samples were mounted using Fluoroshield^TM^ with 4′,6-diamidine-2′-phenylindole dihydrochloride (Sigma-Aldrich^®^, St. Louis, MO, USA). For the acrosome reaction, the presence or absence of the acrosome was evaluated, and nearly a total of 200 cells were analyzed per sample. AR was evaluated in non-capacitated (AR NC) sperm, in capacitated sperm after 1 h and 4 h of in vitro capacitation (AR C 1 h and AR C 4 h), and following AR induction at 1 h and 4 h of capacitation (AR 1 h and AR 4 h).

### 2.6. Immunofluorescence of Tyrosine Phosphorylation

**Figure 1:**
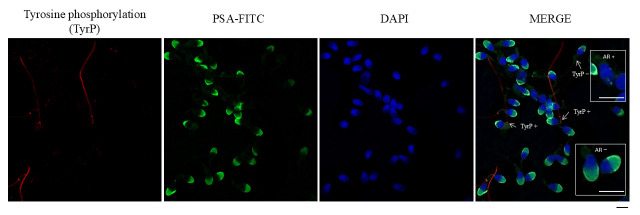
Immunofluorescence analysis of human sperm, showing tyrosine phosphorylation (TyrP), acro-somal status, and nuclear staining. From left to right: TyrP: Red fluorescence, indicating proteins phosphorylated on tyrosine residues, primarily localized in the sperm flagellum. PSA-FITC: Green fluorescence, marking the acrosomal region in mainly acrosome-intact sperm, detected with fluorescein-conjugated Pisum sativum agglutinin (FITC-PSA). DAPI: Blue fluorescence staining the nuclei of sperm. MERGE: Overlay of the three channels, showing co-localization of acrosomal, nuclear, and tyrosine phosphorylation signals. Arrows indicate sperm positive for tyrosine phosphorylation (TyrP+). AR+: acrosome reacted. AR−: acrosome intact. Scale bar: 5 μm.

For each paraformaldehyde-fixed condition, 5 μL of sperm suspension was placed onto a coverslip. Cells were then permeabilized by incubating with 0.1% (*v*/*v*) Triton X-100 (Sigma-Aldrich) for 10 min. Unspecific binding was prevented using 2% (*w*/*v*) BSA-PBS for 30 min. Tyrosine phosphorylation (TyrP) was detected with an anti-phosphotyrosine primary antibody produced in mice (PY20, Sigma-Aldrich^®^, St. Louis, MO, USA) at a 1:500 dilution for 1 h, and a secondary anti-mouse IgG antibody conjugated to Cy3 (Jackson ImmunoResearch, Ely, UK) at a 1:300 dilution (see Figure 1). Finally, coverslips were subsequently mounted with Vectashield and DAPI. For immunofluorescence of tyrosine phosphorylation, the presence or absence of phosphorylation in the sperm flagellum was evaluated, and approximately 200 cells were counted for each preparation, as can be seen in Figure 1. TyrP was evaluated in NC sperm (TyrP NC), and in capacitated sperm after 1 h (TyrP C 1 h) and 4 h (TyrP C 4 h) of in vitro capacitation.

### 2.7. Mitochondrial Membrane Potential Assessment (MMP)

**Figure 2:**
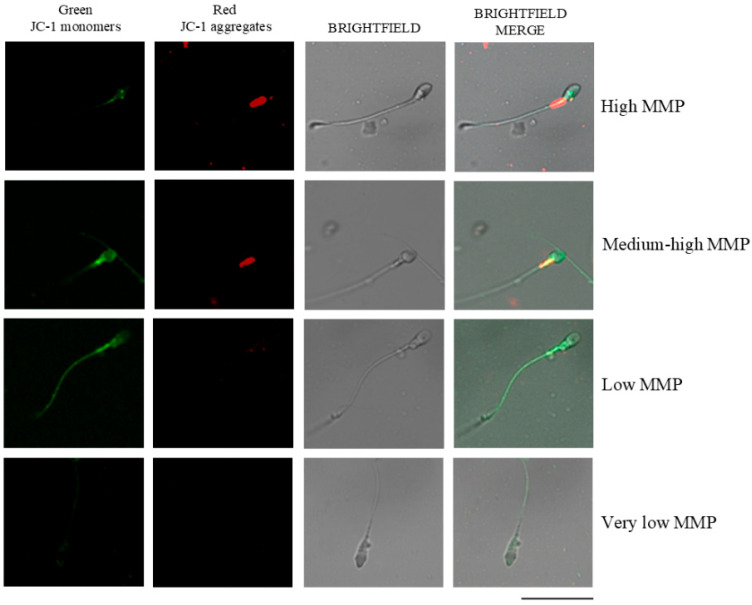
Fluorescence micrographs illustrating mitochondrial membrane potential (MMP) in human sperm. Sperm exhibiting high MMP appear red, those with moderately high MMP appear orange, low MMP sperm appear green, and sperm with very low MMP show no fluorescence. JC-1: 5,5′,6,6′-tetrachloro-1,1′,3,3′-tetraethylbenzimidazolylcarbocyanine iodide. MMP: mitochondrial membrane potential. Adapted and modified from previously published work [25]. Images were acquired using a Zeiss LSM 800 confocal laser scanning microscope (Zeiss, Oberkochen, Germany). Scale bar: 20 μm.

MMP was determined in NC sperm cells using the lipophilic dye JC-1 (5,5′,6,6′-tetrachloro-1,1′,3,3′-tetraethylbenzimidazolcarbocyanine iodide; MERCK, Darmstadt, Germany), following the protocol described by Carrageta et al. [24]. Fresh sperm samples were diluted with PBS to a final concentration of 1 × 10^6^ cells/mL and incubated with JC-1 at 0.6 μg/μL for 20 min at 37 °C in a 5% CO_2_ atmosphere. The MMP was subsequently evaluated using a Leica DM750 fluorescence microscope (Leica, Wetzlar, Germany). A FITC/GFP filter set (excitation 460–490 nm, dichroic mirror > 500 nm, emission > 510 nm) was used. This filter allows for the simultaneous detection of the green (~529 nm) and red (~590 nm) JC-1 emissions, which are perceived together as an orange signal when both forms of the dye are present. To visualize non-fluorescent cells, the fluorescence lamp was switched off, and the same microscopic field was observed under bright-field optics. At least 200 spermatozoa per sample were analyzed based on the color of midpiece fluorescence: high MMP (red), medium/moderately high MMP (orange), low MMP (green), and very low MMP (no fluorescence), according to previous criteria [25] (Figure 2).

### 2.8. Seminal Plasma Samples’ Treatment and Standards

Seminal plasma samples were merely 1:1 diluted in Milli-Q ultrapure water. Note that it has been reported that the method based on sample digestion affords higher limits of detection than the dilution one [17].

A 100 μg/L multielemental standard was prepared (SCP33MS, SCP SCIENCE, Clark Graha, Baie D’Urfé, QC, Canada) and contained the following elements: Ag, Al, B, Ba, Be, Bi, Ca, Cd, Co, Cr, Cu, Fe, K, Li, Mg, Mn, Na, Ni, Pb, Sr, Zn, Tl, As, Sb, Se, U, V, Mo, Ce, La, Rb, Sn, and Ti. To apply internal standardization, a solution containing Sc, Ge, Rh, and Re (SCP SCIENCE, Clark Graha, Baie D’Urfé, Canada) was added online to the main sample stream by using a tee connector prior to the nebulizer of the spectrometer. Table 1 shows the nuclides selected as internal standards for the different analytes.

### 2.9. Instrumentation

Ionic intensities were measured by using an ICP-MS/MS instrument (Agilent 8900 ICP-QQQ, Agilent Technologies, Santa Clara, CA, USA). This instrument is equipped with an octopole collision–reaction cell (CRC) located in between two quadrupole analyzers. Two cell modes (no gas and He) were selected. Moreover, a mixture of argon/oxygen (80/20) was added as optional gas to mitigate carbon deposits.

### 2.10. Statistical Analysis

All statistical analyses were conducted using SPSS version 25 for Windows (SPSS Inc., Chicago, IL, USA). Data normality was first assessed using the Shapiro–Wilk test. Based on these results, Spearman’s correlation coefficients were computed to evaluate the associations among seminal parameters. Statistical significance was set at *p* < 0.05. Heatmaps were generated with GraphPad Prism version 8 (GraphPad Software, San Diego, CA, USA).

Subsequently, samples were stratified into two groups according to the mean concentration of each element in seminal plasma: a low group (values below the mean) and a high group (values above the mean). Differences in sperm quality parameters, mitochondrial membrane potential, acrosome reaction, and tyrosine phosphorylation between groups were assessed using the Kruskal–Wallis test for independent samples.

## 3. Results

### 3.1. Identification of the Conditions Resulting in Optimum Sensitivities and Accurate Results

In order to achieve accurate results, operating conditions should be optimized from the point of both sensitivity and the absence of interferences. The latter point is often neglected, and, in the present work, besides signal optimization, a study about the correction of interferences was undertaken.

#### 3.1.1. Optimization of the Operating Conditions from the Point of View of Sensitivity

The selected variables were (i) the RF power, i.e., the power supplied by the instrument RF generator responsible for the total energy produced to sustain the plasma; (ii) the nebulizer gas flow rate, Q_gneb_, which corresponded to the gas stream used to turn the sample, i.e., diluted human SP, into an aerosol which was finally delivered to the plasma in order to transform the elements into free ions; and (iii) the aerosol phase dilution gas flow rate, Q_gdil_, which was a gas stream added at the plasma torch base. All three variables precluded both the analytical sensitivity achieved and the tolerance of the high-temperature plasma to human SP matrix. The 10 μg/L standard was analyzed together with a blank consisting of ultrapure water.

The impact of Q_gneb_ on the normalized ionic intensity is shown in Figure S1a. Normalized ionic intensity was obtained by dividing each signal by the maximum value found. It is worth noting that four different analytes were initially considered: Li, Mn, Sb, and Pb. These elements have *m*/*z* ratios of 7, 55, 121, and 208, respectively, while their first ionization energies are 5.39, 7.44, 8.64, and 7.42 eV, respectively. Therefore, the observed trends were representative of the overall range of analytes, as this covered the full spectrum of *m*/*z* ratios and ionization energies. Figure S1a clearly shows that the higher the Q_gneb_, the higher the sensitivity up to 1 L/min. Above this gas flow rate, the signal decreased significantly for all four analytes. An increase in Q_gneb_ yields finer aerosols and improves the carrying capability of the gas stream, promoting the transport of solution towards the plasma and improving sensitivity. However, excessively high Q_gneb_ reduced the analyte plasma residence time, leading to decreased ion production and thus lower sensitivity. Additionally, increasing the total central gas load may reduce plasma temperature and ionization efficiency. In order to monitor this latter effect, the signal for the ^36^Ar isotope was also registered. At 1.1 L/min, the signal for this nuclide was around 20% lower than that measured for lower nebulizer gas flow rates (see Figure S1a).

Regarding the plasma RF power (Figure S1b), an increase in this variable led to a maximum in normalized ionic intensity. For Li, Mn, and Pb, the maximum signal was observed at 1400 W. However, in the case of high 1st IP elements, such as Sb, as well as As, Be, and Cd, among others, the optimum RF power slightly shifted to 1500 W. Consequently, 1500 W was selected as the optimal value. The signal for ^36^Ar increased steeply with the RF power, suggesting that the plasma ionization capability improved as this variable rose.

The dilution gas flow rate was also evaluated for its impact on ICP-MS/MS signal. As noted, this corresponded to an argon stream added at the exit of the spray chamber whose main purpose was to dilute the sample matrix in aerosol phase, thereby increasing the tolerance of the instrument to complex matrices. In the present work, Q_gneb_ was maintained at 1 L/min, and the total gas flow (Q_gdil_ + Q_gneb_) was varied from 1.0 (no dilution gas) to 2.2 L/min. It was observed that, for all the analytes tested, the maximum signal was achieved for a Q_gdil_ value of 0.3 L/min.

#### 3.1.2. Recovery Tests Under Sensitivity Optimum Conditions

To test the absence of interferences in the results provided by the ICP-MS/MS instrument, recovery tests were undertaken. In order to perform these studies, five real seminal plasma samples were spiked with the multielemental solution. Then, the following equation was applied:

(1) $$\mathrm{Recovery}=\frac{\left(\mathrm{Intensity}\;\mathrm{spiked}\;\mathrm{sample})\;-(\mathrm{Intensity}\;\mathrm{non}-\mathrm{spiked}\;\mathrm{sample}\right)}{\mathrm{Intensity}\;\mathrm{of}\;\mathrm{aqueous}\;\mathrm{standard}}\times 100$$

According to Equation (1), for a given real sample, ionic intensities were taken for three solutions: (i) the spiked sample at a given multielemental concentration, i.e., 50 μg/L; (ii) the non-spiked sample; and (iii) a standard prepared in ultrapure water, having the same analyte concentration as that added to the sample. A 100% value in recovery indicated that the sample matrix did not affect the value of the analytical signal.

**Figure 3:**
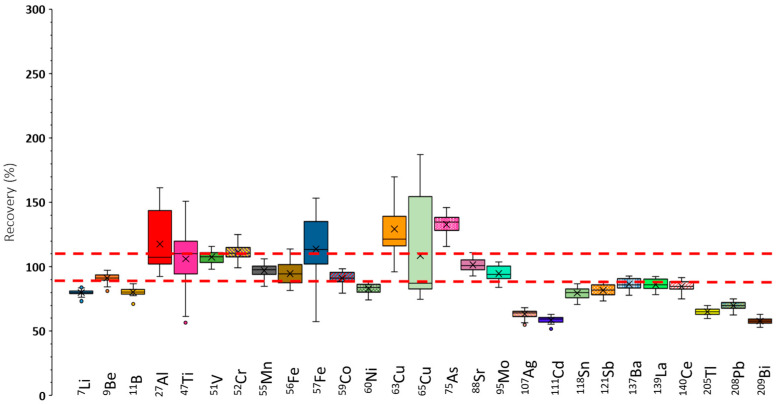
Recoveries found under operating conditions optimum from the point of view of sensitivity. Q_gneb_: 1 L/min. Q_gdil_: 0.3 L/min. RF power: 1500 W. Red dashed lines indicate the recovery acceptance interval (90–110%).

Figure 3 shows the recoveries obtained for the determined elements in a box-and-whiskers diagram. Note that the selected ICP-MS/MS operating conditions correspond to the optimal ones in terms of sensitivity (Figure S1). The points considered were the median of all the data (i.e., the horizontal line inside the box), the 25th and 75th percentiles (lower and upper box limits), and the total range, given by the extreme of the whiskers. Furthermore, outliers were represented as isolated points. To consider a given set of results as valid, a ±10% tolerance, delimited by the dashed red lines in Figure 3, was applied. Overall, the recoveries found under operating conditions leading to maximum sensitivities were not satisfactory. Nevertheless, for nuclides such as ^9^Be, ^51^V, ^55^Mn, ^59^Co, ^88^Sr, and ^95^Mo, recoveries approached 100% according to the acceptability criterion used in the present study. For the remaining elements, recoveries differed significantly from the target value. Therefore, it was concluded that, under these conditions, elements showing poor recoveries suffered from interferences.

Nuclides such as ^27^Al, ^47^Ti, ^52^Cr,^57^Fe, ^63^Cu, ^65^Cu, and ^75^As showed recoveries that, in some cases, exceeded 100%. This could be partially due to the existence of spectral polyatomic interferences caused by the sample matrix, which contained high concentrations of carbon, sodium, potassium, calcium, and magnesium [26], among other elements (e.g., chlorine, sulfur, and phosphorus). Table 2 summarizes representative spectral interferences caused by the presence of these elements, as well as the interfered nuclides. Furthermore, Table 2 also contains polyatomic interferences induced by elements present in the plasma (argon, nitrogen, and oxygen). These results confirmed expectations, and the aforementioned nuclides could overlap with polyatomic ions, leading to an overestimation of their concentration (i.e., recoveries above 100%).

For other nuclides, it was observed that recoveries were significantly lower than 100%. These were ^7^Li, ^11^B, ^60^Ni, and the heaviest nuclides (^107^Ag through ^209^Bi). In this case, non-spectral interferences could explain these findings. Note that high-salt-content solutions may cause a decrease in the sensitivity compared with plain-water standards. Therefore, underestimation of the analyte concentration is expected, leading to recoveries below 100%.

Additionally, it was found that the variability of the recoveries, depending on the sample, was high for some nuclides. Thus, for ^27^Al, ^47^Ti, ^57^Fe, ^63^Cu, and ^65^Cu, recoveries could vary by up to a 100% or even more as a function of the particular sample analyzed. This fact demonstrated a high impact of the specimen on the accuracy of the method.

Because of the degraded recoveries shown in Figure 3, two different methodologies were employed to overcome matrix effects: (i) internal standardization and (ii) the use of a collision gas. To apply the former methodology, a solution containing 50 μg/L of four different elements was used for internal standardization. The elements were germanium (Ge), scandium (Sc), rhodium (Rh), and rhenium (Re). The two former elements were used to compensate for non-spectral interferences produced on light masses, whereas the two latter ones, were applied to heavier isotopes (see Table 1). To compensate for spectral interferences, a helium (He) stream was added to the ICP-MS/MS octopole collision/reaction cell. This approach was applied because it provides a universal solution, since He dissociates the bonds created in polyatomic ions, hence leaving the analyte free of spectral interferences. The obtained recoveries are shown in Figure S2.

Globally speaking, the results in Figure S2 show improvement, as compared to Figure 3, because the recoveries were closer to 100%. Exceptions to this rule were ^7^Li, ^9^Be, ^75^As, ^88^Sr, and ^95^Mo, for which the data deviated more markedly from 100% than when neither internal standardization nor collision gas had been applied. Additionally, it was observed that, for most nuclides, at least a fraction of the samples yielded recoveries outside the tolerance level. The range of recoveries found for ^11^B, ^27^Al, ^57^Fe, and ^65^Cu was excessively wide. This fact suggested that, again, the recovery depended strongly on the sample used in this study. It became evident that ICP-MS/MS operating conditions producing maximum sensitivities were not suitable for accurate multielemental analysis of human SP. Therefore, different plasma settings were selected.

#### 3.1.3. Recovery Tests Under Robust ICP-MS/MS Conditions

The basic idea was to try to enhance the plasma robustness, thereby enabling more efficient ionization of different analytes regardless of the matrix composition. The optimization involved increasing the plasma RF power from 1500 to 1600 W and suppressing the dilution gas stream. To verify that the plasma energy increased, in addition to monitoring the ^36^Ar signal, the ^9^Be/^7^Li ratio was evaluated. The results demonstrated that the higher the RF power, the higher this ratio. Thus, ^9^Be/^7^Li went from 0.12 to 0.17 as this variable increased from 1.2 to 1.6 kW. Meanwhile, an increase in the total gas flow rate led to a significant drop in the mentioned ratio (from 0.25 to 0.004 when switching from 1 to 2.2 L/min). Notably, at 0.3 L/min, the ratio was close to 0.17.

**Figure 4:**
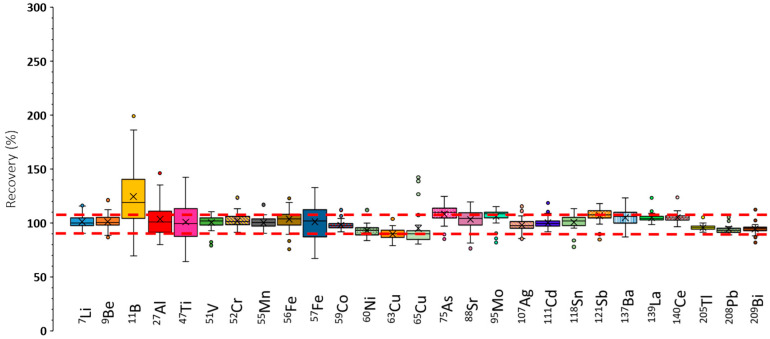
Recoveries found under operating conditions leading to a robust plasma, applying internal standardization and adding He to the collision/reaction octopole cell (Q_gHe_: 3 mL/min). Q_gneb_: 1 L/min. Q_gdil_: 0 L/min. RF power: 1600 W. Red dashed lines indicate the recovery acceptance interval (90–110%).

The recoveries achieved under these circumstances are presented in Figure S3. When compared with the results shown in Figure 3, it can be concluded that recoveries were substantially improved by operating the plasma under robust conditions This effect was particularly evident for elements such as ^75^As and ^95^Mo, for which recoveries approached 100%. Nonetheless, further improvement was achieved by applying internal standardization and using He as collision gas. The corresponding data, illustrated in Figure 4, indicate that recoveries were consistently close to 100%, with narrow error bars, demonstrating that recovery was independent of the sample matrix for most of the nuclides analyzed.

#### 3.1.4. Limits of Detection and Quantification Under Robust ICP-MS/MS Conditions

The best recovery results were obtained under robust plasma conditions. However, this improvement in accuracy was accompanied by a decrease in sensitivity. Specifically, signals under robust conditions were 5-fold lower (e.g., ^7^Li) to more than 10-fold lower (e.g., ^55^Mn, ^121^Sb, and ^208^Pb) compared with those achieved under conditions for maximum sensitivity (Figure S1). Nevertheless, the recovery tests confirmed that robust conditions were essential to ensure accurate determination. To further assess the analytical performance, an additional study was conducted to evaluate the effect of He as a collision gas on the ICP-MS/MS detection capabilities. Limits of detection (LODs) and quantification (LOQs) were calculated according to the following equations:

(2) $$LOD=\frac{3\;s_{b}}{m}$$

(3) $$LOQ=\frac{10\;s_{b}}{m}$$

where *s_b_* is the standard deviation (SD) of ten consecutive blank measurements, and *m* is the slope of the calibration line.

In the first place, it was observed that, with a few exceptions, the use of He in the octopole cell increased the LOQ values by a factor between 1.5 and 13 under high-sensitivity conditions. In contrast, under robust plasma conditions, the effect of the collision gas differed (Table 3). In general, LOQs calculated in the presence of He in the collision/reaction cell were comparable, or even lower than those obtained in its absence. For certain isotopes (e.g., ^56^Fe), the absence of a collision gas resulted in a relatively high background signal, which accounted for the elevated LODs observed.

### 3.2. Andrology Results

#### 3.2.1. Characteristics of This Study’s Population, Semen Analysis Results, and Sperm Biomarkers

Data about the 29 semen donors of the seminal samples included in this study are summarized in Table 4. The donors were aged between 18 and 49 years, with a mean value of 25.28 ± 6.60 years, and with a BMI of 23.90 ± 2.85 kg/m^2^.

#### 3.2.2. Concentrations of Elements in Seminal Plasma

The analysis of real samples was carried out under robust plasma conditions combined with internal standardization. To minimize spectral interferences, ^47^Ti, ^52^Cr,^56^Fe, ^63^Cu and ^75^As were quantified in gas mode, whereas ^51^V, ^55^Mn, ^88^Sr, ^95^Mo, and ^208^Pb were determined under the non-gas mode. Table 5 presents the concentration ranges of the quantified elements found in seminal plasma, together with the proportion of samples in which concentrations were above LOD and LOQ.

#### 3.2.3. Correlations of Heavy Metals and Metalloids Concentrations with Seminal Parameters and Spermatic Biomarkers

**Figure 5:**
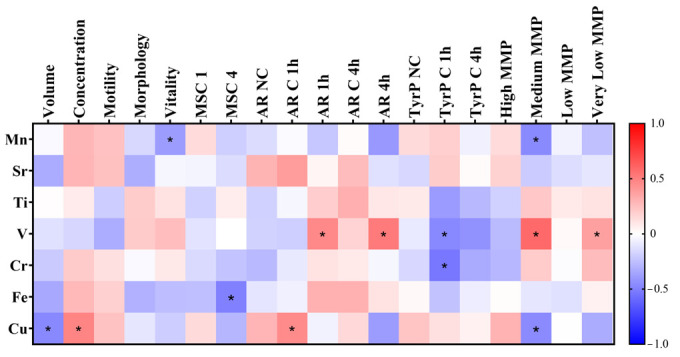
Heatmap showing the correlations found between the trace elements found in SP and sperm parameters. Asterisks (*) show significant results (*p* < 0.05). MSC: motile sperm concentration. NC: non-capacitated. C 1 h: capacitated after 1 h of in vitro capacitation. C 4 h: capacitated after 4 h of in vitro capacitation. AR 1 h: acrosome reacted after 1 h of capacitation. AR 4 h: acrosome reacted after 4 h of capacitation. TyrP: tyrosine phosphorylation. MMP: mitochondrial membrane potential.

Concerning seminal parameters and the elemental concentration of SP, Figure 5 depicts a heatmap of the correlations identified in this study, with asterisks indicating statistical significance. Several interesting associations were observed. For instance, Cu concentration was negatively correlated with sample volume (r = −0.468, *p* = 0.010) and positively correlated with sperm concentration (r = 0.478, *p* = 0.009). Mn levels were negatively correlated with sperm vitality (r = −0.391, *p* = 0.036), while Fe concentration was negatively associated with motile sperm concentration after 4 h of in vitro capacitation (r = −0.496, *p* = 0.022). Regarding sperm biomarkers, Cu concentration showed a positive correlation with the percentage of cells undergoing spontaneous AR after 1 h of in vitro capacitation (r = 0.452, *p* = 0.031). V levels in SP were positively correlated with the proportion of acrosome-reacted sperm after AR induction once seminal samples were in vitro capacitated for 1 h (r = 0.462, *p* = 0.035) and 4 h (r = 0.515, *p* = 0.035). With regard to tyrosine phosphorylation, both V and Cr concentrations were negatively correlated with the percentage of cells exhibiting tyrosine phosphorylation after 1 h of capacitation (r = −0.465, *p* = 0.039; r = −0.537, *p* = 0.015, respectively).

Finally, our results indicated that specific trace elements were correlated with the spermatic mitochondrial membrane potential (MMP). V levels in SP were positively correlated with the percentage of sperm cells with very low MMP (r = 0.370, *p* = 0.048) and medium MMP (r = 0.585, *p* = 0.001). However, Mn and Cu concentrations were negatively correlated with the percentage of sperm cells with medium MMP (r = −0.459, *p* = 0.012, r = −0.458, *p* = 0.013, respectively).

#### 3.2.4. Comparison of Sperm Parameters Between High and Low Element Levels in Seminal Plasma

Significant differences were observed between groups with element concentrations above and below the mean for several semen parameters and sperm biomarkers (Table S1). Men with lower SP Mn concentrations exhibited a significantly higher proportion of sperm with medium MMP compared with men with higher SP Mn levels (4.52 ± 4.24% vs. 0.86 ± 1.41%, *p* = 0.034).

Regarding V, men with lower SP V concentrations demonstrated reduced sperm viability (83.66 ± 9.27% vs. 89.65 ± 5.14%, *p* = 0.025), as well as a lower percentage of acrosome-reacted sperm after AR induction at 1 h (38.72 ± 20.10% vs. 65.78 ± 22.67%, *p* = 0.015) and 4 h (51.32 ± 23.36% vs. 72.11 ± 16.03%, *p* = 0.043) of in vitro capacitation. This group also showed a lower proportion of sperm with medium MMP (1.09 ± 2.13% vs. 5.36 ± 4.13%, *p* = 0.001) and very low MMP (12.94 ± 13.92% vs. 23.84 ± 11.88%, *p* = 0.016), while exhibiting a higher percentage of tyrosine phosphorylation in capacitated sperm after 1 h of in vitro capacitation (21.35 ± 12.61% vs. 11.05 ± 12.18%, *p* = 0.041).

For Fe, men with lower SP Fe concentrations had a significantly higher semen volume (4.00 ± 1.24 mL vs. 2.98 ± 0.90 mL, *p* = 0.020), but a lower sperm concentration (79.80 ± 43.39 million/mL vs. 133.63 ± 76.50 million/mL, *p* = 0.027) and reduced motility (62.92 ± 15.02% vs. 73.15 ± 20.33%, *p* = 0.030).

Finally, men with lower SP Cu concentrations exhibited a higher semen volume (3.99 ± 1.02 mL vs. 2.90 ± 0.90 mL, *p* = 0.018), together with a lower proportion of sperm undergoing spontaneous acrosome reaction in non-capacitated cells (14.86 ± 10.87% vs. 22.22 ± 12.65%, *p* = 0.037) and after 1 h of in vitro capacitation (9.83 ± 6.73% vs. 18.98 ± 9.22%, *p* = 0.023). No significant differences were observed for Sr, Ti, and Cr.

## 4. Discussion

The present study provides, to our knowledge, the first comprehensive characterization of trace elements in SP using ICP-MS/MS under robust plasma conditions. From a methodological standpoint, this approach demonstrated that operating the instrument under robust plasma settings, combined with helium introduction into the collision/reaction cell, was critical to minimizing background signals and ensuring accurate, reproducible determinations. This methodology enabled the reliable quantification of multiple trace elements (Mn, Sr, Ti, V, As, Cr, Fe, Cu, Mo, and Pb), most of which were detected above the LOD and LOQ in the majority of samples. Importantly, the concentrations of several elements were significantly associated with key semen parameters and sperm functional biomarkers related to male fertility. These findings indicate that specific trace elements in SP may influence sperm function at multiple levels—from basic seminal quality parameters to molecular markers of capacitation and oxidative stress—underscoring their potential role in male fertility.

### 4.1. Novel ICP-MS/MS Analysis Method

The use of a method based on merely diluting the sample and subsequently introducing it into the spectrometer is straightforward compared to procedures based on acid sample digestion at high temperatures. The latter methodologies can lead to multiple errors due to the complexity of the experimental protocol, sample contamination by elements that may be present in the reagents used, or the loss of some elements that generate volatile species throughout the sample preparation process. Furthermore, the LOQs achieved by diluting the sample dilution by a factor of 2 are lower than those achieved using acid digestion procedures, in which the final SP dilution factor can be as high as 1:20 or even higher. This fact leads to the possibility of detecting elements that, until now, could not be quantified or had been measured at concentrations close to the LOQ. The lower dilution factor applied in the method developed in the present work permitted detecting ten trace elements in SP. Among them, Mn, Sr, Ti, V, Cr, Fe, and Cu were encountered in virtually all the analyzed samples (Table 5), whereas As, Mo, and Pb were only in a low fraction of SP, giving a proof of their absence in most of the samples.

Obviously, applying a dilution and shot analysis procedure ensured that the matrix remained intact and its organic components were not chemically degraded, which required an effective and reliable signal measurement system. In this area, the ICP-MS/MS methodology stands out, as it makes it possible to mitigate non-spectral interferences, since it is possible to employ an auxiliary matrix effect correction technique, such as the online internal standard method. In this regard, the recovery results obtained through doping of real samples (Figure 4) led to the conclusion that, by using ICP-MS/MS equipment, it was possible to eliminate such non-spectroscopic interferences. Indeed, the matrix components of the diluted samples affect the processes of sample introduction into the plasma (nebulization and aerosol transport).

Furthermore, the sample concomitants can lead to plasma degradation effects, because these components may take a given amount of energy from the plasma. As a consequence, the available energy for analyte ionization decreased, thus providing lower sensitivities. This effect led to a degradation of the trueness of the final results obtained. In order to overcome plasma thermal degradation, robust conditions had to be applied. These circumstances corresponded to a low total central gas flow rate (Q_gdil_ + Q_gneb_) and high plasma RF power. Because of the enhanced amount of energy, the plasma improved its tolerance to the sample matrix and decomposed it more efficiently, and the performance of the system became independent of the sample composition. More reliable analytical results were obtained (see Figure 4).

Finally, PS matrix components caused spectroscopic polyatomic interferences (Table 2) that led to an overestimation of the obtained analyte concentrations. In this regard, the ICP-MS/MS technique eliminated interfering ions by using a collision gas such as He. This solution was adopted in the present study because of its universality and applicability to any type of interfering polyatomic ion. Thus, the final recovery values approached 100% in the presence of this He in the equipment’s octupole (Figure 4).

Taking into account all of the abovementioned comments, the analytical survey of the ICP-MS/MS instrument for the characterization of SP using a dilution and single-shot analysis approach revealed that, to obtain accurate results, the instrument must be operated under robust plasma conditions. Under these conditions, the plasma showed increased tolerance to the SP biological matrix compared with maximum-sensitivity settings. This optimization was essential for the multielement determination of such samples, as operating under high-sensitivity settings often produces unrealistic results. These methodological considerations are frequently overlooked, leading to errors in reported concentrations. Interestingly, under robust plasma conditions, the addition of helium to the collision/reaction cell was beneficial for LOD and LOQ values, as background signals were markedly reduced. Under these optimized conditions, the ICP-MS/MS method achieved sufficiently low LOQs to provide reliable information on ten analytes, supporting the recommendation to use robust plasma settings with helium.

### 4.2. Relationship of Heavy Metals and Metalloids in Seminal Plasma with Seminal Parameters and Sperm Biomarkers

The results indicated that seminal plasma contained Cu, Fe, Mn, Sr, and Ti in all samples, while V and Cr were quantified in most cases. Notably, elements such as Cu, Mn, and Cr may contribute to a pro-oxidative microenvironment that favors ROS generation.

#### 4.2.1. Impact of Manganese

Mn concentrations were consistent with previous studies, with a mean of 6.6 μg/L in SP from men with normal seminal parameters [28]. Although Mn is an essential element, excessive levels may be harmful [29]. The present study demonstrated that Mn negatively affected sperm vitality and mitochondrial membrane potential (MMP), as it correlated inversely with the percentage of sperm cells exhibiting medium MMP. Previous reports indicated higher Mn levels in SP from abnormal compared with normal groups [30]. Elevated serum Mn has also been associated with impaired sperm motility and morphology in healthy men without occupational exposure [31], as well as with increased sperm DNA damage in urine biomonitoring studies [32]. Nevertheless, trace amounts of Mn are crucial for normal sperm function [33], as Mn is a cofactor of metalloenzymes such as Mn-superoxide dismutase (Mn-SOD), located in the mitochondrial matrix. In this context, antioxidant enzymes protect cells from excessive ROS production [34].

#### 4.2.2. Impact of Strontium

Sr concentrations measured in this study were lower than those reported elsewhere, where values reached 126.9 ± 75.4 μg/L in normozoospermic men and 145.6 ± 79.9 μg/L in non-normozoospermic patients [35]. Recent evidence suggests that strontium exposure may benefit semen quality, as urinary Sr levels have been positively associated with sperm concentration, motility, and total count [36].

#### 4.2.3. Impact of Titanium

Ti was detected at several tens of μg/L in all samples, whereas higher concentrations were reported in other studies [35]. Ti is widely used in cosmetics, household products, and food, making exposure difficult to avoid. TiO_2_ nanoparticles (TiO_2_NPs) may migrate into seminal plasma, raising toxicological concerns. Experimental models have shown that TiO_2_NPs can induce genotoxicity, cytotoxicity, genome instability, apoptosis, and reduced cell viability in vertebrates [37,38,39]. Moreover, TiO_2_NPs have been linked to DNA damage in sperm, likely mediated by intracellular ROS production [40].

#### 4.2.4. Impact of Iron

Fe is considered a non-enzymatic antioxidant present in SP [41]. It exerts protective effects against oxygen-derived toxic products but can also act as a pro-oxidant, catalyzing the formation of highly reactive hydroxyl radicals from hydrogen peroxide and superoxide. These ROS are especially damaging to sperm plasma membranes due to their high content of polyunsaturated fatty acids. Fe also plays an essential role in spermatogenesis and male fertility [42]. Pathological elevations in Fe can induce sperm damage via ROS generation and lipid peroxidation. Similar to our results, Fe has been reported to negatively affect sperm morphology [43], and in vitro studies showed that Fe^2+^ reduces sperm motility through lipid peroxidation [44]. Moreover, Fe correlates negatively with glutathione (tGSH) levels, suggesting depletion of antioxidant defenses. In contrast, Fe-binding proteins such as lactoferrin can mitigate ROS generation at physiological levels. Previous studies also reported differences in Fe concentrations between normozoospermic and non-normozoospermic men [28], with higher Fe levels in patients with abnormal semen parameters [45]. In this study, Fe concentrations were positively correlated with motile sperm after 4 h of in vitro capacitation.

#### 4.2.5. Impact of Copper

Cu was quantified in all SP samples. As an essential trace element, it serves as a cofactor for metalloenzymes, including Cu/Zn–superoxide dismutase (Cu/Zn-SOD), where Cu^2+^ plays a catalytic role. However, ionic copper can rapidly become toxic by promoting ROS generation through the Fenton and Haber–Weiss reactions, leading to protein oxidation, enzyme inactivation, and structural damage. Sperm are particularly vulnerable due to their high content of polyunsaturated fatty acids. Elevated Cu levels have been associated with increased total oxidative status (TOS) in SP and with malondialdehyde (MDA), a lipid peroxidation marker. High Cu concentrations have been linked to idiopathic oligoasthenoteratozoospermia, suggesting an amplification of oxidative damage [46,47]. Conversely, Cu deficiency can reduce ejaculate volume, sperm count, motility, and morphology [47]. In this study, SP Cu concentrations correlated positively with sperm concentration but negatively with the proportion of sperm cells exhibiting medium MMP. This aligns with previous evidence associating high Cu levels with impaired motility and vitality [48].

#### 4.2.6. Impact of Chromium

Cr was found in all samples at concentrations above the LOD. At elevated levels, Cr can disrupt metabolism and promote ROS generation [23]. Associations have been described between SP Cr concentrations and progressive motility, total sperm count, and sperm concentration. However, Cr exposure has also been linked to increased DNA damage [27]. Specifically, Cr, together with Cu and Zn, has been associated with increased comet assay tail length and distributed tail moment in highly exposed individuals, indicating sperm DNA damage. The concentrations measured here were consistent with previous reports [27].

#### 4.2.7. Impact of Arsenic, Molybdenum, and Lead

A relatively low percentage of samples contained As and Mo (≈56%), while Pb was detected in very few cases. As is a metalloid and potentially toxic element (PTE) that adversely affects male reproduction [48]. It induces sperm germ cell apoptosis through stress-dependent pathways, contributing to infertility [49]. Reported As concentrations vary widely, from 1.4 μg/L [28] to >30 μg/L [50,51,52]. As exposure increases ROS and MDA levels while reducing GSH and SOD activity, leading to impaired antioxidant defenses [8]. Mo exposure has been linked to reproductive toxicity in animals and humans [52,53,54], including reduced sperm concentration and abnormal morphology [55]. In this study, Mo was detected in 55.2% of samples but was quantified in only three, precluding correlation analyses.

Pb is a well-known endocrine disruptor [55,56] associated with male infertility. High Pb accumulation in semen has been linked to reduced sperm counts in non-occupationally exposed men [56]. Pb concentrations are elevated in infertile versus fertile men and correlate negatively with progressive motility while positively with DNA fragmentation and ROS levels [57]. Pb induces oxidative stress by increasing MDA and reducing antioxidant enzymes such as SOD and glutathione peroxidase [6]. Moreover, Pb can deplete sulfhydryl (-SH) groups due to its high thiol affinity, lowering glutathione levels and increasing susceptibility to ROS-mediated damage [58,59].

#### 4.2.8. Biological Implications of the Results

Our findings suggest that seminal plasma trace elements influence key aspects of sperm function. For instance, Cu and Mn concentrations were negatively correlated with sperm mitochondrial membrane potential (MMP) and vitality, indicating that these elements may modulate oxidative stress (OS) and mitochondrial activity, processes essential for sperm motility and overall functional integrity. Similarly, V, Cu, and Cr levels correlated with tyrosine phosphorylation and acrosome reaction, both of which are critical for capacitation and fertilization.

Group-based analyses further supported these associations. Comparisons between samples with higher versus lower seminal plasma concentrations of trace elements revealed significant differences in conventional semen parameters and sperm functional biomarkers. Specifically, variations in Mn, V, Fe, and Cu were associated with changes in semen volume, sperm motility and concentration, mitochondrial activity, and the capacity of sperm to undergo acrosome reaction or tyrosine phosphorylation. These results highlight that the balance of seminal plasma trace elements may regulate multiple facets of sperm function, underscoring their potential role in male reproductive health.

These observations are consistent with previous evidence that human semen represents a sensitive biomarker of environmental exposure to metals and other contaminants [60]. Importantly, elemental concentrations differ between semen and blood, emphasizing the need to analyze multiple biological matrices to capture systemic exposure comprehensively. Moreover, inter-individual variability in element levels supports the idea that intrinsic factors such as age, diet, and environmental conditions may modulate these associations.

Collectively, these findings indicate that even subtle alterations in elemental concentrations could affect male fertility by modifying both conventional semen parameters and functional sperm biomarkers. This emphasizes the importance of future studies involving larger and geographically diverse cohorts, multiple biological matrices, and longitudinal designs to elucidate the role of environmental and potentially toxic elements in oxidative stress, sperm function, and reproductive health.

### 4.3. Limitations of This Study

The present study has several limitations that should be acknowledged. First, the sample size was relatively small (*n* = 29), with most participants being normozoospermic and only two cases of asthenozoospermia. Thus, larger studies including men with impaired sperm quality are required to confirm and expand the present observations. Second, certain potentially toxic elements (PTEs), such as As, Pb, and Mo, were below the detection limit or detected in only a few samples, restricting correlation analyses. Accordingly, the absence of significant associations should be interpreted with caution, as it may reflect methodological or sampling limitations rather than the absence of biological effects.

Third, the cross-sectional design, with element levels and sperm parameters assessed at a single time point, precludes causal inference. Moreover, biological variability should be considered, since trace element concentrations may fluctuate over time and a single sample may not fully capture individual exposure. Finally, only seminal plasma was analyzed; evaluation of additional biological matrices (e.g., blood and urine) would provide complementary information on systemic exposure.

Taken together, these limitations highlight the need for future studies with larger and more diverse cohorts, longitudinal designs, and multi-matrix approaches. Although significant correlations were identified, they should be interpreted cautiously, as correlation does not imply causation. Further work is required to elucidate the underlying mechanisms.

## 5. Conclusions

The present study underscores the importance of an accurate analytical methodology for characterizing the multielement profile of human seminal plasma and highlights the complex correlations between trace elements and biomarkers of male reproductive health, including their impact on oxidative stress balance. Accurate multielemental analysis using ICP-MS/MS requires operation under robust plasma conditions, combined with internal standardization and helium (He) as a collision gas. These conditions are crucial to mitigate the complex effects of the biological matrix and eliminate spectroscopic interferences, although they may lead to lower sensitivity compared to other configurations. Notably, under robust conditions, the addition of He in the collision/reaction cell can further improve detection and quantification limits by reducing background noise.

Regarding the results found for human SP real samples, they were highly relevant because the presence of ten different chemical elements was confirmed. Thus, elements such as Cu, Mn, and Cr can contribute to a pro-oxidative seminal microenvironment that promotes ROS generation. Excess Cu and Fe can act as pro-oxidants, inducing oxidative stress and damage to sperm membranes due to their high polyunsaturated fatty acid content, in addition to depleting important antioxidants such as glutathione. Toxic elements such as As and Pb also induce oxidative stress by increasing ROS levels and decreasing the activity of antioxidant enzymes. In contrast, Mn at trace levels is crucial for normal sperm function, acting as a cofactor for antioxidant enzymes such as superoxide dismutase, which protects against the damaging effects of excess ROS. To our knowledge, no previous data are available regarding the presence of V in seminal plasma.

Specific correlations between elemental concentrations and seminal quality parameters suggest that these elements may serve as potential biomarkers for male fertility assessment. The consistent detection of Ti in all seminal plasma samples is particularly relevant, given that TiO_2_ nanoparticles have been reported to exert genotoxic and cytotoxic effects, potentially compromising sperm DNA integrity through oxidative stress-mediated pathways. Future studies should incorporate additional functional sperm biomarkers and mechanistic analyses to better elucidate the reproductive impact of trace elements—particularly Ti—on male fertility.

## Figures and Tables

**Table 1:** Operating conditions of ICP-OES and ICP-MS/MS.

Parameter	Value
RF power (kW)	1.6
Plasma flow (L/min)	15
Auxiliary flow (L/min)	1
Nebulizer flow (L/min)	0.4
Makeup gas (L/min)	0.3
Spray chamber temperature (°C)	−5
Sampling depth (mm)	8
Liquid flow (μL/min)	100
Scan type	MS/MS
Replicates	6
Collision reaction gas/flow rate (mL/min)	He/3.0
Stabilization time (s)	No gas: 10 He: 20
Sweeps	10
Internal standards	Analytes
^45^Sc	^7^Li, ^9^Be, ^11^B, ^27^Al, ^47^Ti, ^51^V, ^52^Cr, ^55^Mn, ^56^Fe, ^57^Fe
^72^Ge	^59^Co, ^60^Ni, ^63^Cu, ^65^Cu, ^75^As
^103^Rh	^88^Sr, ^95^Mo, ^107^Ag, ^111^Cd, ^118^Sn, ^121^Sb, ^137^Ba, ^139^La, ^140^Ce
^185^Re	^205^Tl, ^208^Pb, ^209^Bi

**Table 2:** Potential spectral interferences for seminal plasma samples.

Nuclide	Abundance	Interfering Polyatomic Ion
^27^Al	100	^12^C^15^N^+^, ^13^C^14^N^+^, ^14^N_2_^+^, n^1^H^12^C^14^N^+^
^47^Ti	7.32	^32^S^14^N^1^H^+^, ^32^S^15^N^+^, ^33^N^14^N^+^, ^33^S^14^N^+^, ^15^N^16^O_2_^+^, ^14^N^16^O_2_^1^H^+^, ^12^C^35^Cl^+^, ^31^P^16^O^+^
^52^Cr	83.76	^35^Cl^16^O^1^H^+^, ^40^Ar^12^C^+^, ^36^Ar^16^O^+^, ^37^Cl^15^N^+^, ^34^S^18^O^+^, ^36^S^16^O^+^, ^38^Ar^14^N^+^, ^36^Ar^15^N^1^H^+^, ^35^Cl^17^O^+^
^56^Fe	91.66	^40^Ar^16^O^+^, ^40^Ca^16^O^+^, ^40^Ar^15^N^1^H^+^, ^38^Ar^18^O^+^, ^38^Ar^17^O^1^H^+^, ^37^Cl^18^O^1^H^+^
^57^Fe	2.19	^40^Ar^16^O^1^H^+^, ^40^Ca^16^O^1^H^+^, ^40^Ar^17^O^+^, ^38^Ar^18^O^1^H^+^
^60^Ni	26.16	^44^Ca^16^O^+^, ^23^Na^37^Cl^+^, ^43^Ca^16^O^1^H^+^
^63^Cu	69.1	^31^P^16^O_2_^+^, ^40^Ar^23^Na^+^, ^47^Ti^16^O^+^, ^23^Na^40^Ca^+^, ^46^Ca^16^O^1^H^+^, ^36^Ar^12^C^14^N^1^H^+^, ^14^N^12^C^37^Cl^+^, ^16^O^12^C^35^Cl^+^
^65^Cu	30.9	^32^S^16^O_2_^1^H^+^, ^40^Ar^25^Mg^+^, ^40^Ca^16^O^1^H^+^, ^36^Ar^14^N^21^H^+^, ^32^S^33^S^+^, ^32^S^16^O^17^O^+^, ^33^S^16^O_2_^+^, ^12^C^16^O^37^Cl^+^, ^12^C^18^O^35^Cl^+^, ^31^P^16^O^18^O^+^
^75^As	100	^40^Ar^35^Cl^+^, ^36^Ar^38^Ar^1^H^+^, ^38^Ar^37^Cl^+^, ^36^Ar^39^K, ^43^Ca^16^O_2_, ^23^Na^12^C^40^Ar, ^12^C^31^P^16^O_2_^+^

Adapted from Ref. [27].

**Table 3:** Limits of detection (LODs) and quantification (LOQs) obtained with and without collision gas under conditions leading to a robust plasma.

	LOD (μg/L)		LOQ (μg/L)	
Analyte	He Gas *	No Gas	He Gas *	No Gas
^47^Ti	2.36	2.98	7.85	9.92
^51^V	0.16	0.09	0.54	0.32
^52^Cr	0.52	1.05	1.73	3.49
^55^Mn	0.63	0.41	2.1	1.36
^56^Fe	1.64	63.43	5.48	211.45
^63^Cu	9.51	6.63	31.70	26.09
^75^As	1.71	0.66	5.69	2.21
^88^Sr	0.28	0.26	0.94	0.87
^95^Mo	1.70	1.07	5.67	3.58
^208^Pb	0.20	0.29	0.67	0.96

* He was introduced in the octopole cell at 3 mL/min.

**Table 4:** Demographic, expositional, seminal and sperm’s biomarker data.

	Mean ± SEM	Min	Max
Demographic data			
Age (years)	25.28 ± 6.60	18.00	49.00
BMI (kg/m^2^)	23.90 ± 2.85	18.51	29.98
Expositional data			
Occupationally exposed	5 (17.24%)		
Environmentally exposed	0		
Smoker	8 (27.59%)		
Seminal data			
Volume (mL)	3.54 ± 1.20	1.00	7.00
Sperm concentration (mill/mL)	103.93 ± 67.12	19.50	327.67
Sperm motility (P +NP) (%)	67.51 ± 18.02	18.90	96.00
Sperm morphology (%)	8.29 ± 2.37	4.00	13.21
Sperm viability (%)	86.35 ± 8.16	55.00	99.07
MSC 1 h (mill/mL)	19.36 ± 20.07	0.40	74
MSC 4 h (mill/mL)	10.15 ± 6.45	2.7	22.6
Sperm biomarkers			
Acrosome reaction			
AR NC	17.53 ± 11.82	2.00	51.00
AR C 1 h	13.41 ± 8.86	3.00	30.00
AR 1 h	52.90 ± 25.11	16.00	95.20
AR C 4 h	11.28 ± 8.13	2.00	26.87
AR 4 h	62.33 ± 21.95	19.12	95.00
Tyrosine phosphorylation			
TyrP NC	5.85 ± 7.35	0.00	28.00
TyrP C 1 h	16.20 ± 13.17	2.00	41.10
TyrP C 4 h	18.37 ± 12.91	1.94	41.00
Mitochondrial activity			
High MMP (%)	57.01 ± 15.13	26.79	90.90
Medium MMP (%)	3.00 ± 3.79	0	13.46
Low MMP (%)	22.16 ± 17.49	4.00	59.80
Very low MMP (%)	17.83 ± 13.96	0	48.15

MSC: motile sperm concentration. NC: non-capacitated. C 1 h: capacitated after 1 h of in vitro capacitation. C 4 h: capacitated after 4 h of in vitro capacitation. AR 1 h: acrosome reacted after 1 h of capacitation. AR 4 h: acrosome reacted after 4 h of capacitation. TyrP: tyrosine phosphorylation. MMP: mitochondrial membrane potential.

**Table 5:** Trace elements’ concentrations in μg/L found in real seminal plasma samples.

Analyte	Mean ± SEM	Min	Max	Samples Above LOD (%)	Samples Above LOQ (%)
^55^Mn	5.23 ± 2.5	1.41	11.76	100	100
^88^Sr	74.29 ± 31.93	28.09	166.36	100	100
^47^Ti	567.42 ± 2474.52	5.55	13,156.53	100	100
^51^V	1.13 ± 0.45	<LOQ	2.52	100	96.55
^75^As	10.84 ± 11.22	<LOD	30.90	58.62	17.24
^52^Cr	5.55 ± 15.34	<LOQ	84.99	100	93.10
^56^Fe	126.93 ± 86.13	47.53	497.89	100	100
^63^Cu	88.58 ± 41.18	31.09	206.09	100	100
^95^Mo	1.78 ± 0.96	<LOD	3.34	55.17	10.34
^208^Pb	0.37 ± 0.88	<LOD	4.92	6.90	3.57

## Supplementary Materials

The following supporting information can be downloaded at https://www.mdpi.com/article/10.3390/antiox14091118/s1. Figure S1: Normalized ionic intensity with respect to the maximum signal versus (a) the nebulizer gas flow rate; dilution gas flow rate, Q_gdil_ = 0.3 L/min; RF power, 1600 W and (b) RF power; dilution gas flow rate, Q_gdil_ = 0.3 L/min; nebulizer gas flow rate, Q_gneb_ = 1.0 L/min. Figure S2: Recoveries found under operating conditions optimum from the point of view of sensitivity by applying internal standardization and adding He to the collision/reaction octopole cell (Q_gHe_: 3 mL/min). Q_gneb_: 1 L/min. Q_gdil_: 0.3 L/min. RF power: 1500 W. Figure S3: Recoveries found under operating conditions leading to a robust plasma. Q_gneb_: 1 L/min. Q_gdil_: 0 L/min. RF power: 1600 W. Table S1: Comparison of sperm parameters between high and low levels of trace elements in seminal plasma. Kruskal–Wallis test results for Mn, Sr, V, Fe, Cu, Ti, and Cr are shown.
