# Supplementary material for: Multielemental Profile for Seminal Plasma Through Inductively Coupled Plasma–Tandem Mass Spectrometry and Its Relationship with Seminal Parameters, Spermatic Biomarkers, and Oxidative Stress
Source: Antioxidants (Basel). 2025 Sep 15;14(9):1118. doi: 10.3390/antiox14091118 (PMC12466358; doi:10.3390/antiox14091118)
Supplement: Supplementary file 1 [file antioxidants-14-01118-s001.zip › antioxidants-3824014-supplementary.pdf]

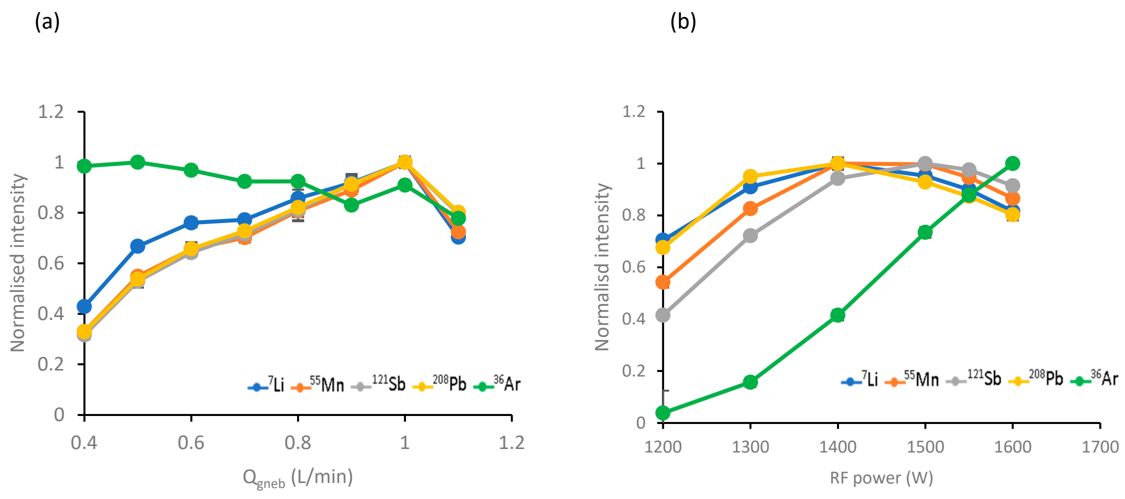

Figure S1. Normalised ionic intensity with respect to the maximum signal versus (a) the nebulizer gas flow rate; dilution gas flow rate, Q_{gdil} = 0.3 L/min; RF power: 1600 W and (b) RF power; dilution gas flow rate, Q_{gdil} = 0.3 L/min; nebulizer gas flow rate, Q_{gneb} = 1.0 L/min.

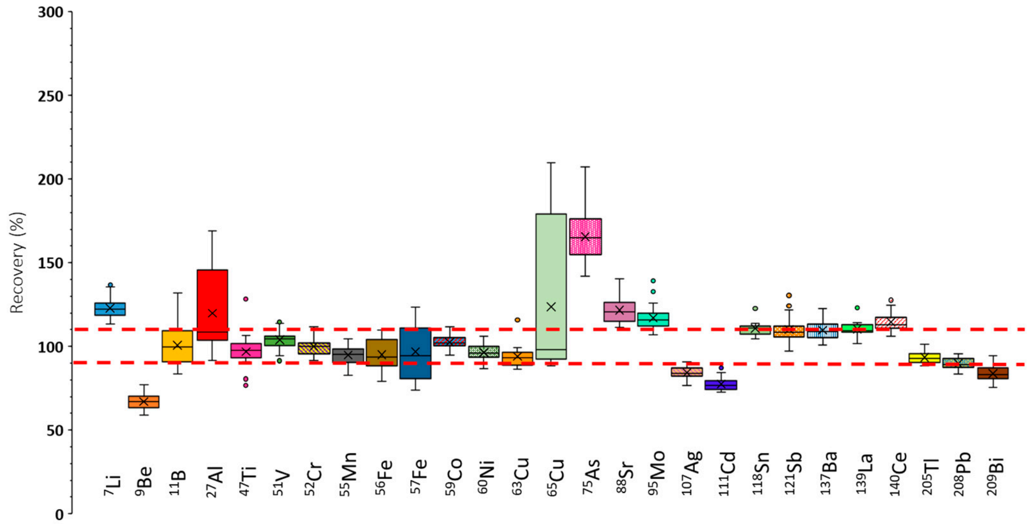

Figure S2. Recoveries found under operating conditions optimum from the point of view of sensitivity by applying internal standardization and adding He to the collision/reaction octopole cell (Q_{gHe}: 3 mL/min). Q_{gneb}: 1 L/min; Q_{gdil}: 0.3 L/min; RF power: 1500 W.

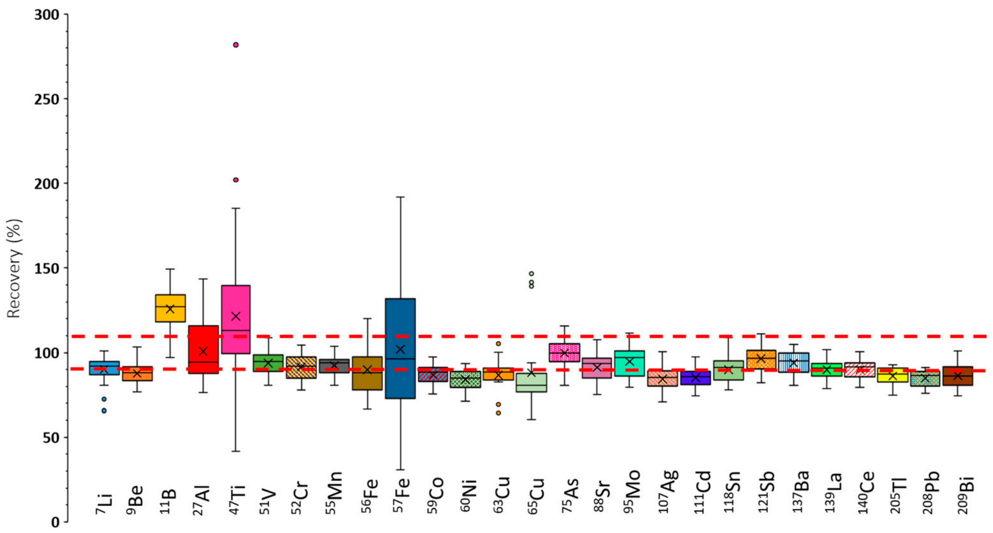

Figure S3. Recoveries found under operating conditions leading to a robust plasma. Q_{gneb}: 1 L/min; Q_{gdil}: 0 L/min; RF power: 1600 W.

Table S1: Comparison of sperm parameters between high and low levels of trace elements in seminal plasma. Kruskal–Wallis test results for Mn, Sr, V, Fe, Cu, Ti, and Cr are shown.

Element	Parameter	H	df	p-value
**Mn**	Volume (mL)	0.018	1	0.894
	Sperm concentration (mill/mL)	0.012	1	0.912
	Sperm motility (P +NP) (%)	0.096	1	0.757
	Sperm morphology (%)	0.059	1	0.807
	Sperm viability (%)	2.074	1	0.150
	MSC 1h (mill/mL)	0.385	1	0.535
	MSC 4h (mill/mL)	0.317	1	0.573
	AR NC	0.517	1	0.472
	AR C 1h	0.001	1	0.974
	AR 1h	1.344	1	0.246
	AR C 4h	0.058	1	0.810
	AR 4h	2.086	1	0.149
	TyrP NC	0.374	1	0.541
	TyrP C 1h	1.062	1	0.303
	TyrP C 4h	0.054	1	0.817
	High MMP (%)	0.567	1	0.452
	Medium MMP (%)	7.098	1	**0.008**
	Low MMP (%)	0.031	1	0.859
	Very low MMP (%)	2.136	1	0.144
**Sr**	Volume (mL)	1.279	1	0.258
	Sperm concentration (mill/mL)	1.263	1	0.261
	Sperm motility (P +NP) (%)	1.314	1	0.252
	Sperm morphology (%)	1.167	1	0.280

	Sperm viability (%)	0.656	1	0.418
	MSC 1h (mill/mL)	0.008	1	0.928
	MSC 4h (mill/mL)	0.336	1	0.562
	AR NC	0.798	1	0.372
	AR C 1h	1.050	1	0.306
	AR 1h	0.062	1	0.804
	AR C 4h	1.053	1	0.305
	AR 4h	0.153	1	0.696
	TyrP NC	1.401	1	0.237
	TyrP C 1h	0.013	1	0.908
	TyrP C 4h	1.681	1	0.195
	High MMP (%)	0.517	1	0.472
	Medium MMP (%)	0.744	1	0.388
	Low MMP (%)	0.396	1	0.529
	Very low MMP (%)	0.146	1	0.702
**V**	Volume (mL)	0.438	1	0.508
	Sperm concentration (mill/mL)	0.000	1	0.983
	Sperm motility (P +NP) (%)	0.770	1	0.380
	Sperm morphology (%)	1.020	1	0.313
	Sperm viability (%)	5.009	1	**0.025**
	MSC 1h (mill/mL)	0.002	1	0.965
	MSC 4h (mill/mL)	0.045	1	0.831
	AR NC	1.330	1	0.249
	AR C 1h	3.532	1	0.060
	AR 1h	5.906	1	**0.015**
	AR C 4h	0.188	1	0.665
	AR 4h	4.088	1	**0.043**
	TyrP NC	0.443	1	0.506
	TyrP C 1h	4.169	1	**0.041**
	TyrP C 4h	1.929	1	0.165
	High MMP (%)	0.433	1	0.511
	Medium MMP (%)	11.332	1	**0.001**
	Low MMP (%)	1.848	1	0.174
	Very low MMP (%)	5.819	1	**0.016**
**Fe**	Volume (mL)	5.371	1	**0.020**
	Sperm concentration (mill/mL)	4.907	1	**0.027**
	Sperm motility (P +NP) (%)	4.714	1	**0.030**
	Sperm morphology (%)	2.430	1	0.119
	Sperm viability (%)	0.809	1	0.368
	MSC 1h (mill/mL)	0.277	1	0.598
	MSC 4h (mill/mL)	0.972	1	0.324
	AR NC	0.004	1	0.947
	AR C 1h	0.001	1	0.975

	AR 1h	1.429	1	0.232
	AR C 4h	2.230	1	0.135
	AR 4h	0.002	1	0.962
	TyrP NC	0.406	1	0.524
	TyrP C 1h	2.039	1	0.153
	TyrP C 4h	1.929	1	0.165
	High MMP (%)	0.377	1	0.539
	Medium MMP (%)	0.207	1	0.649
	Low MMP (%)	1.508	1	0.219
	Very low MMP (%)	0.192	1	0.661
**Cu**	Volume (mL)	5.581	1	**0.018**
	Sperm concentration (mill/mL)	3.712	1	0.054
	Sperm motility (P +NP) (%)	0.412	1	0.521
	Sperm morphology (%)	0.826	1	0.363
	Sperm viability (%)	0.059	1	0.807
	MSC 1h (mill/mL)	0.000	1	0.982
	MSC 4h (mill/mL)	0.727	1	0.394
	AR NC	4.366	1	**0.037**
	AR C 1h	5.156	1	**0.023**
	AR 1h	0.159	1	0.690
	AR C 4h	0.000	1	1.000
	AR 4h	0.647	1	0.421
	TyrP NC	1.510	1	0.219
	TyrP C 1h	0.430	1	0.512
	TyrP C 4h	0.000	1	1.000
	High MMP (%)	1.129	1	0.288
	Medium MMP (%)	3.461	1	0.063
	Low MMP (%)	0.384	1	0.535
	Very low MMP (%)	0.237	1	0.626
**Ti**	Volume (mL)	0.882	1	0.348
	Sperm concentration (mill/mL)	1.647	1	0.199
	Sperm motility (P +NP) (%)	0.375	1	0.540
	Sperm morphology (%)	2.215	1	0.137
	Sperm viability (%)	0.031	1	0.861
	MSC 1h (mill/mL)	0.003	1	0.953
	MSC 4h (mill/mL)	0.393	1	0.531
	AR NC	0.117	1	0.733
	AR C 1h	0.026	1	0.871
	AR 1h	1.062	1	0.303
	AR C 4h	1.253	1	0.263
	AR 4h	2.453	1	0.117
	TyrP NC	0.353	1	0.552
	TyrP C 1h	0.429	1	0.513

	TyrP C 4h	0.021	1	0.885
	High MMP (%)	0.412	1	0.521
	Medium MMP (%)	0.336	1	0.562
	Low MMP (%)	0.340	1	0.560
	Very low MMP (%)	1.042	1	0.307
**Cr**	Volume (mL)	0.378	1	0.539
	Sperm concentration (mill/mL)	0.093	1	0.760
	Sperm motility (P +NP) (%)	0.285	1	0.593
	Sperm morphology (%)	2.254	1	0.133
	Sperm viability (%)	1.431	1	0.232
	MSC 1h (mill/mL)	0.622	1	0.430
	MSC 4h (mill/mL)	0.552	1	0.457
	AR NC	1.582	1	0.208
	AR C 1h	0.044	1	0.833
	AR 1h	0.171	1	0.680
	AR C 4h	1.555	1	0.212
	AR 4h	0.157	1	0.692
	TyrP NC	0.481	1	0.488
	TyrP C 1h	2.335	1	0.126
	TyrP C 4h	1.091	1	0.296
	High MMP (%)	2.990	1	0.084
	Medium MMP (%)	2.200	1	0.138
	Low MMP (%)	0.003	1	0.959
	Very low MMP (%)	2.817	1	0.093

In bold p-value <0.05. MSC: motile sperm concentration, NC: non-capacitated, C 1h: capacitated after 1h of in vitro capacitation, C 4h: capacitated after 4h of in vitro capacitation, AR 1h: acrosome reacted after 1 h of capacitation, AR 4h: acrosome reacted after 4 h of capacitation, TyrP: Tyrosine phosphorylation, MMP: mitochondrial membrane potential.
